# Running demands in club, regional, national, and international provincial New Zealand rugby union competitions

**DOI:** 10.3389/fspor.2022.1062043

**Published:** 2023-01-13

**Authors:** Peter Olsen, Richard Deuchrass, Shaun Owen, Matt Lilley, James Jowsey, Michael Hamlin

**Affiliations:** ^1^ Department of Applied Sciences and Social Practice, Ara Institute of Canterbury, Christchurch, New Zealand; ^2^ Department of Tourism, Sport and Society, Lincoln University, Lincoln, New Zealand; ^3^ Canterbury Rugby Union, Christchurch, New Zealand

**Keywords:** GPS technology, running, conditioning, loading, rehabilitation, team sports

## Abstract

The demands of national and international professional rugby union matches are well established, however, there has not been a comparative study investigating running demands in New Zealand teams playing in club (amateur), Heartland Championship (semi-professional Div 2), the Mitre 10 Cup (semi-professional Div 1) or Super Rugby (professional) competitions. This information could enable specific training and rehabilitation that programmes to be developed to meet the needs of players in the different competitions. Players wore 10 Hz GPS units during games for one rugby season to determine absolute (m) and relative (m.min^−1^) measures for total distance, running volume (∼≥7 km·h^−1^) and high intensity running (∼≥16 km·h^−1^). There were typically minimal differences (1–2 m.min^−1^) in running distance measures between amateur level front row forwards and inside backs compared to players in these positions at higher levels of competition. Therefore, amateur players in these positions may find the transition to higher competitions less challenging with respect to running load. In contrast, amateur outside backs and back row forwards may find the increased pace of higher levels of competition more challenging due to typically covering significantly less running and high intensity running distances in amateur games. Differences for half backs were more variable between the levels of competition. Based on our results, it cannot be assumed that amateur rugby has lower running demands than higher competitions or that there is a continuum of increased running demands with increasing competition levels, as some playing positions in the semi-professional (Div 2) (second lowest level of competition) team recorded the largest values for total distance, running and high intensity running. Therefore, the specificity of running demands in a position and competition need to be considered individually for each player when transitioning between competitions. The practice and perception of returning a professional player to amateur club rugby due to the belief that running loads being lower may also be flawed, as we found considerable positional variation in running demands within-and-between competitions.

## 1. Introduction

Rugby union is a collision team sport played by two teams over two 40-min halves. Players are required to perform intermittent low to high intensity activity including static efforts, collisions, walking, jogging, and sprinting in a game (1, 2). Since the introduction of professionalism in rugby union in 1995, there has been substantial research investigating the movement characteristics and physical demands of rugby in elite male players. For over a decade, there has also been considerable use of Global Positioning Systems (GPS) to measure the running demands in professional rugby (2–4).

Global Positioning Systems are a satellite-based technology that tracks movements over time and are widely used in team sports to measure player positions and speed (2, 4). This technology allows for the intensity of activity and physiological load placed on players to be monitored during trainings and games (1, 2). Collected data can be used by trainers and coaches to plan and implement programmes that elicit physiological adaptations specific to the demands of a game (2, 5). There has been extensive research determining the physical demands of rugby in professional male players using time and motion analysis, and GPS (2, 6–14). A study by Jones et al. (5) using GPS found professional European male rugby players typically covered between 3698 m to 6436 m during a game depending on position. On average, tight forwards (i.e., front and second row forwards) covered 3698 m to 5027 m, loose forwards (flankers and number-eight) 4868 m to 5741 m, inside backs (halves and centres) 4987 m to 6086 m, and outside backs (wingers and fullback) 6181 m to 6436 m. Backs also typically covered greater distances at higher speeds during a game compared to forwards. A similar study by Dubois et al. (15) on professional male players found backs covered 300 m to 800 m at higher speeds (>14.4 km·h^−1^) and in sprinting (>25 km·h^−1^) whereas forwards covered more distance in the moderate speed zones (10–14.4 km·h^−1^). A recent review by Bridgeman and Gill (2) of running demands in age grade, academy and senior professional rugby players also found that on average backs covered greater total, relative and high-speed running distances than forwards. However, while there is considerable research on elite academy and professional players, there has been limited research on amateur club and semi-professional players who make up a large proportion of the rugby union playing population.

Recent research by King et al. (16) found New Zealand amateur male club level players covered similar total distances as those reported for elite rugby players. However, when data was separated into back or forward positions, distances were typically lower than those reported in the literature for the elite players. These findings indicate there might be differences in the running demands at different levels of competition in specific positions (16–18). In contrast, a recent study by Takamori et al. (19) found there was minimal difference in running demands when Club players data was compared to previous research on professional players, when data was expressed in relative terms as distance covered per minute (m.min^−1^). A limitation of previous research is that there has not been an examination of the running demands across amateur, semi-professional and professional matches in different competitions. In New Zealand, there are amateur club competitions within provinces, competitions between provinces including the Mitre 10 Heartland Championship (semi-professional Div 2) and the Mitre 10 Cup (professional Div 1), and the Investec Super Rugby (professional) competition between teams from New Zealand, Australia, and South Africa (20). Information on the running demands in these competitions could increase trainers’ and coaches’ understanding of possible differences in running loads between different levels of competition, assist in the transition of players between competitions, and in the management of training loads when a player is returning to play after injury (2, 21). Consequently, we examined the running demands in New Zealand rugby at amateur, semi-professional, and professional levels of competition.

## 2. Materials and methods

### 2.1. Research design

This study used a retrospective research design to determine the running demands of different positions in four teams in Club (amateur), Mitre 10 Heartland Championship (semi-professional Div 2), Mitre 10 Cup Premiership (semi-professional Div 1) and Investec Super Rugby (professional) competitions in a rugby season. All teams were located in the same geographical or provincial region of New Zealand. No attempt was made to control for variables such as weather or the win: loss profile of teams.

### 2.2. Participants

Data collection for the amateur and semi-professional (Div 2) games occurred in the 2017 rugby season, while data from the semi-professional (Div 1) and professional games was collected in the 2019 season. Each team had a squad of approximately 24 players. All players were familiarized with the devices as part of their normal training and playing practices. In total, there were 602 data points collected over the season, where one data point represents a player who has completed at least 60 min of playing time in a game. Data was collected from the amateur team in 17 games (203 data points), the semi-professional (Div 2) team for 9 games (83 data points), the semi-professional (Div 1) team for 11 games (138 data points), and the professional team for 16 games (178 data points). The study had Institutional Ethics Board approval and subjects were informed of the benefits and risks of the investigation prior to signing an institutionally approved informed consent document to participate in the study.

### 2.3. Procedures

#### 2.3.1. GPS equipment and measures

Individualized 10-Hz GPS units (Viper pod 2, STATSports [amateur, semi-professional (Div 1) and professional teams], Belfast, UK, or VX Sport^™^ (semi-professional (Div 2) were placed inside a tight-fitting vest or in a pocket in a player's jersey in the region of the upper thoracic spine. Units were turned on approximately 10 min prior to a game. After each match, GPS data were downloaded using the manufacturer's software package and subsequently cropped (to include match play data only) for further analysis. The GPS measurements collected were total distance (m), meters per minute (m.min^−1^), running distance (∼≥7 km·h^−1^) and high intensity running (∼≥16 km·h^−1^) as previously used in New Zealand Super Rugby research (12). These running measures were used to enable a comparison of data from the different teams, as there was a lack of consistency in speed zones used by the teams in the field, and also in the literature (2, 4, 12, 22). For example, for the running distance measurement, there was a range of speeds used in the field (7.0 km·h^−1^to 7.3 km·h^−1^), therefore the running distance measurement throughout the article is referred to as ∼≥7 km·h^−1^. Relative distance measurements (meters per minute, m.min^−1^) were calculated by dividing total distance by minutes played.

### 2.4. Player positions

To examine the differences in performance measures between playing positions, the data files were categorized broadly into forwards (hooker, prop, lock, loose forwards, number-8) and backs (half back, first five, second five, centre, wing and fullback). Similar to previous research (7), we also categorized the players into more specific playing positions to account for their unique roles within the team including front row (prop/hooker), second row (lock), back row (flankers/number-8), half back, inside backs (first five/second five/centre) and outside backs (wings/fullback). The number of data points for specific positions in the amateur, semi-professional (Div 2) semi-professional (Div 1) and professional teams were respectively; front row (*n* = 35, 15, 21, 19), second row (*n* = 27, 17, 18, 24), back row (*n* = 39, 14, 29, 39), half back (*n* = 13, 5, 12, 8), inside backs (*n* = 46, 14, 28, 49) and outside backs (*n* = 43, 18, 30, 39).

### 2.5. Statistical analysis

To examine the differences in performance measures between playing positions, the data files were categorized as described above in subheading 1.4. Group means and standard deviations were calculated for total distance (m), running (∼≥7 km·h^−1^) distance (m), and high intensity (≥16.1 km·h^−1^) running (m) for the four levels of competition (amateur, semi-professional (Div 2), semi-professional (Div 1) and professional). Meters per minute (m.min^−1^) for each measure were calculated by dividing total distance by minutes played. Differences in the mean of the variables and standard deviations representing the between and within-subject variability were estimated using a mixed modelling procedure (Proc Mixed) in the Statistical Analysis System (Version 9.3, SAS Institute, Cary North Carolina, USA). *P* values were produced for the between group comparisons and we used an alpha level of *p* ≤ 0.05 for significance in this study. This study used a convenience sample of players from four teams over one rugby season as described in subheading 1.1 and 1.2.

## 3. Results

### 3.1. Absolute and relative distance measurements in different rugby competitions

#### 3.1.1. Total distance (m) and running distance (∼≥7 km·h^−1^)

On average, there was a trend for lower competition [amateur and semi-professional (Div 2)] players to cover greater absolute distances (∼150 m to ∼400 m) per game compared to players in higher levels of competition [semi-professional (Div 1) and professional] players (Table 1). This difference was significant for semi-professional (Div 2) data compared to all levels of competition (*p* < 0.05), and for amateur vs. professional level (*p* = 0.05). A similar trend was observed in relative m.min^−1^ data (*p* < 0.05, Table 1). As in total distance measures, there was also a trend for semi-professional (Div 2) players to run more in games (∼120 m) compared to other levels of competition, with this difference being significant (*p* < 0.01) when expressed in relative m.min^−1^ e.g., 42 m.min^−1^ vs. ∼39 m.min^−1^ for the semi-professional (Div 2) players vs. other competition players respectively (Table 1). In contrast, amateur level players typically covered less absolute and relative running distance than other competition players (3102 m vs. 3237 m to 3319 m: ∼38 m.min^−1^ vs. 39 to 42 m.min^−1^, *p* = 0.10 to *p* < 0.01).

**Table 1:** Absolute and relative distance measurements (mean ± SD) in different levels of rugby competition.

Competition Level	Absolute total distance (m)	Relative total distance (m.min^−1^)	Absolute distance ∼≥7 km·h^−1^ (m)	Relative distance ∼≥7 km·h^−1^ (m.min^−1^)	Absolute distance ≥16 km·h^−1^ (m)	Relative distance ≥16 km·h^−1^ (m.min^−1^)
Amateur	5746 ± 853^S^	70 ± 7^S^	3102 ± 566^II^^,^^S^	38 ± 6.0^S^	728 ± 351^L^	9 ± 4^L^
Semi-professional (Div 2)	6010 ± 985^L^	76 ± 11^L^	3319 ± 804	42 ± 10^L^	867 ± 426	11 ± 5
Semi-professional (Div 1)	5649 ± 1150	68 ± 10	3239 ± 772	39 ± 8	910 ± 414	11 ± 5
Professional	5548 ± 987	68 ± 8	3237 ± 634	40 ± 7	962 ± 360	12 ± 4

Data are mean ± SD. Superscript letters indicate significant change (*p* ≤ 0.05) between competition levels.

^L^ Significantly different compared to all other levels.

^II^ Significantly different to semi-professional (Div 2).

^S^ Significantly different to professional.

#### 3.1.2. High intensity running (≥16 km·h^−1^)

In contrast to other measures, the highest-level team (professional) covered the most distance at high intensity (∼≥16 km·h^−1^) with players typically running 962 m or 11.7 m.min^−1^ per game at high intensity (Table 1). Whereas amateur level players covered significantly less distance at high intensity compared to all other levels (728 m and 9 m.min^−1^, *p* = 0.10 to *p* < 0.01).

### 3.2. Absolute and relative measurements for forwards and backs

#### 3.2.1. Total distance for backs and forwards

There was a tendency for backs at lower levels [e.g., amateur and semi-professional (Div 2)] to cover more total distance during games compared to players in the professional level of competition (Table 2). This difference was significant (*p* < 0.05) for semi-professional (Div 2) players compared to all other levels for relative distance covered, and for amateur players vs. professional players (Table 2). A similar trend was observed for forwards with lower-level forwards covering significantly (*p* < 0.05) more relative total distance than higher-level forwards (Table 2).

**Table 2:** Absolute and relative distance measurements (mean ± SD) for forward and back positions in different levels of rugby competition.

Position and Level	Absolute total distance (m)	Relative total distance (m.min^−1^)	Absolute distance ∼≥7 km·h^−1^ (m)	Relative distance ∼≥7 km·h^−1^ (m.min^−1^)	Absolute distance ≥16 km·h^−1^ (m)	Relative distance ≥16 km·h^−1^ (m.min^−1^)
**Backs**						
Amateur	6209 ± 707	75 ± 5^S^	3344 ± 503^I^^,^^S^	41 ± 6^L^	989 ± 207^L^	12 ± 3^L^
Semi-professional (Div 2)	6455 ± 933^S^	84 ± 10^L^	3538 ± 720	46 ± 10^L^	1159 ± 337	15 ± 4
Semi-professional (Div 1)	6249 ± 1015	74 ± 8	3633 ± 665	43 ± 6	1199 ± 290	14 ± 3
Professional	6029 ± 844	73 ± 8	3563 ± 537	43 ± 7	1193 ± 252	14 ± 4
**Forwards**						
Amateur	5279 ± 724^S^	65 ± 5^L^	2858 ± 52	35 ± 5	464 ± 257^L^	6 ± 3^L^
Semi-professional (Div 2)	5651 ± 88^L^	71 ± 7^L^	3143 ± 832^L^	39 ± 9^L^	640 ± 344	8 ± 4
Semi-professional (Div 1)	5030 ± 937	62 ± 8	2833 ± 658	35 ± 7	611 ± 294	7 ± 3
Professional	4985 ± 835	62 ± 5	2856 ± 518	36 ± 4	692 ± 269	9 ± 3^I^

Data are mean ± SD. Superscript letters indicate significant change (*p* ≤ 0.05) between competition levels for positional groups.

^L^ Significantly different compared to all other levels.

^I^ Significantly different to semi-professional (Div 1).

^II^ Significantly different to semi-professional (Div 2).

^S^ Significantly different to professional.

#### 3.2.2. Running Distance (∼≥7 km·h^−1^) for backs and forwards

In contrast to total distance measurements, amateur level backs covered less absolute and relative running distance compared to the higher levels of competition (*p* < 0.05). Whereas the semi-professional (Div 2) backs still covered significantly (*p* < 0.05) more relative distance than amateur and higher levels of competition (Table 2). Semi-professional (Div 2) forwards also covered significantly (*p* ≤ 0.01) more absolute and relative running distance compared to all other levels of competition, whereas amateur, semi-professional (Div 1) and professional forwards covered similar distances [semi-professional (Div 2): 3143 m and 39 m.min^−1^ vs. other competition levels: ∼2850 m and ∼35 m.min^−1^, Table 2].

#### 3.2.3. High intensity running distance (≥16 km·h^−1^) for backs and forwards

The amateur level backs and forwards also ran significantly (*p* < 0.01) less distances at high intensities than the higher-level backs and forwards (Table 2). Overall, the professional and semi-professional (Div 1 and Div 2) backs covered similar absolute and relative distances (∼1184 m and ∼14.5 m.min^−1^, Table 2). In contrast, the professional forwards typically covered more distance at higher speeds than the amateur and semi-professional (Div 1) level forwards, with this difference being significant (*p* < 0.05) for the relative measures (9 ± 3 vs. 6 ± 3 and 7 ± 3 m.min^−1^ respectively, Table 2).

### 3.3. Relative measurements for forward and back positions different levels of competition

Table 3 shows lower level [amateur and semi-professional (Div 2)] players covered more relative total distance than higher level competition players for front and second row positions (*p* < 0.01), and semi-professional (Div 2) players covered more total distance than higher competition levels in all forward positions (*p* < 0.01). Semi-professional (Div 2) second and back row positions also covered more (*p* < 0.05) relative running distance (∼≥7 km·h^−1^ (m.min^−1^)) compared to all other levels, whereas there were minimal or non-significant differences for front row distances between the levels (Table 3). There was a tendency for amateur forwards to cover significantly less distance at high intensity running [≥16 km·h^−1^ (m.min^−1^)] compared to higher levels of competition. These differences were significant for amateur back row forwards (*p* < 0.01) compared to all other levels, and amateur front row players vs. semi-professional (Div 1) and professional players (*p* ≤ 0.01), and amateur second rowers vs. semi-professional (Div 2) and professional players (*p* < 0.01). In contrast, semi-professional (Div 2), semi-professional (Div 1), and professional forward players covered similar relative distances for high intensity running. The exception was semi-professional (Div 1) second rowers who covered significantly (*p* < 0.01) less high intensity running distance (2–3 m.min^−1^) than the professional and semi-professional (Div 2) players (Table 3).

**Table 3:** Relative distance measures (mean ± SD) for forward positions in all levels of rugby competition.

Position and level	Distance (m.min^−1^)	∼≥7 km·h^−1^ (m.min^−1^)	≥16 km·h^−1^ (m.min^−1^)
**Front Row**			
Amateur	64 ± 5^I^^,^^S^	35 ± 5	3 ± 2^I^^,^^S^
Semi-professional (Div 2)	66 ± 6^I^^,^^S^	34 ± 6	4 ± 2
Semi-professional (Div 1)	60 ± 8	34 ± 7	5 ± 3
Professional	59 ± 5	35 ± 5	5 ± 3
**Second Row**			
Amateur	65 ± 5^I^^,^^II^	36 ± 4^I^^,^^II^	6 ± 2^II^^,^^S^
Semi-professional (Div 2)	72 ± 7^L^	42 ± 7^L^	9 ± 2
Semi-professional (Div 1)	60 ± 7	30 ± 5^S^	6 ± 2^II^^,^^S^
Professional	62 ± 4	35 ± 4	8 ± 2
**Back Row**			
Amateur	65 ± 4	35 ± 5^I^	8 ± 3^L^
Semi-professional (Div 2)	73 ± 7^L^	42 ± 10^L^	11 ± 3
Semi-professional (Div 1)	65 ± 7	39 ± 6	10 ± 2
Professional	63 ± 4	36 ± 4	10 ± 2

Data are mean ± SD. Superscript letters indicate significant change (*p* ≤ 0.05) between competition levels for positional groups.

^L^ Significantly different compared to all other levels.

^I^ Significantly different to semi-professional (Div 1).

^II^ Significantly different to semi-professional (Div 2).

^S^ Significantly different to professional.

Semi-professional (Div 2) backs typically covered greater relative total distances (*p* < 0.01) than all other competition levels (Table 4). An exception was between semi-professional (Div 2) and amateur inside backs where there was minimal difference (2 m.min^−1^) for the relative total distance covered. Semi-professional (Div 2) halfbacks covered significantly more distance running (5–16 m.min^−1^) than other levels (*p* < 0.01). Semi-professional (Div 2) outside backs also covered more running distance than other levels of competitions, with these differences being significant relative to professional and amateur players (4 m.min^−1^ and 9 m.min^−1^, *p* = 0.03 to *p* < 0.01, respectively). There were minimal non-significant differences (0–1 m.min^−1^) in running distances for inside backs in the different levels of competition (Table 4). Similarly, there were minimal differences (1 m.min^−1^) for high intensity distances covered by inside backs in the different competitions, the exception being a significant (*p* = 0.01) difference between amateur vs. professional players (12 m.min^−1^ vs. 14 m.min^−1^ respectively). Semi-professional (Div 1) and amateur half backs covered significantly less high intensity distance (5–7 m.min^−1^, *p* < 0.01) relative to semi-professional (Div 2) and professional players (Table 4). Amateur outside backs also covered significantly less high intensity running distance than other levels of competition (3–5 m.min^−1^, *p* < 0.01). Whereas there was minimal difference (1–2 m.min^−1^) between the higher levels of competition, although the difference between semi-professional (Div 2) vs. professional outside backs (16 m.min^−1^ vs. 14 m.min^−1^ respectively) was significant (*p* = 0.01).

**Table 4:** Relative distance measures (mean ± SD) for back positions in all levels of rugby competition.

Position and level	Distance (m.min^−1^)	∼≥7 km·h^−1^ (m.min^−1^)	≥16 km·h^−1^ (m.min^−1^)
**Half**			
Amateur	79 ± 5^S^	49 ± 4^S^	15 ± 3^II^^,^^S^
Semi-professional (Div 2)	94 ± 14^L^	61 ± 7^L^	20 ± 1^I^
Semi-professional (Div 1)	76 ± 7^S^	45 ± 6	14 ± 3^S^
Professional	85 ± 7	56 ± 6	21 ± 4
**Inside**			
Amateur	75 ± 5^S^	42 ± 4	12 ± 3^S^
Semi-professional (Div 2)	77 ± 9^I^^,^^S^	41 ± 9	13 ± 5
Semi-professional (Div 1)	72 ± 8^S^	41 ± 7	13 ± 2
Professional	69 ± 6	41 ± 4	14 ± 3
**Outside**			
Amateur	73 ± 5	37 ± 5^L^	11 ± 2^L^
Semi-professional (Div 2)	86 ± 6^L^	46 ± 8^S^	15 ± 3^S^
Semi-professional (Div 1)	75 ± 7	44 ± 6	16 ± 4
Professional	74 ± 8	42 ± 7	14 ± 3

Data are mean ± SD. Superscript letters indicate significant change (*p* ≤ 0.05) between competition levels for positional groups.

^L^ Significantly different compared to all other levels.

^I^ Significantly different to semi-professional (Div 1).

^II^ Significantly different to semi-professional (Div 2).

^S^ Significantly different to professional.

## 4. Discussion

### 4.1. Total distance and running measures for different competition levels

A general trend was observed where the lower-level teams [amateur and semi-professional (Div 2)] covered more total distance during games compared to the higher-level teams [semi-professional (Div 1) and professional]. In contrast, the amateur team (lowest level of competition) typically covered significantly less high intensity running distances compared to the higher levels of competition. While the semi-professional (Div 2) team (second lowest level), generally recorded significantly higher relative running distance measures compared to semi-professional (Div 1) and professional teams (highest levels of competition respectively) and had similar high intensity running values as these higher level teams. This finding indicates there is a not a continuum of running demands from the lowest to highest level of competition, as the second lowest team recorded some of the highest running measurements.

The range of absolute distances (5548 m to 6010 m) covered by the amateur, semi-professional and professional rugby players in this study were similar to those reported in other research on amateur and professional rugby players (2, 5, 12, 19). In contrast, the relative total distances found in this study (68–76 m.min^−1^) were considerably higher than those found by King (16) for New Zealand club level (amateur) rugby players (62 m.min^−1^). The reason for the higher values in our research is not known but could be due to methodological differences, in our research we used data from players who had played 60 min or more, whereas King may not have used a threshold for minimal game time. Therefore, the lower values in the King et al. study (16) may be due to differences in the amount of time played (19).

Absolute and relative data in Tables 1, 2 sometimes indicated significant differences for absolute distance measures but not relative distance metrics (and vice versa). Relative measures of distance are probably the most valid measure of running performance in a research setting as they enable valid comparisons between datasets and previous research, as the measures account for differences in time played in different positions and competitions (19). However, absolute measures of running performance may be more useful and easier to use in the field to determine the running load experienced by a player and to inform conditioning decisions. For example, a substitute player who only played for a short period of time in a game, may need to do a top-up run at the end of a rugby game to make certain they have experienced the typical running load for their position in a game. This ensures the player has had a sufficient training load, which could be important in terms of a players’ periodization programme and/ or managing the conditioning of the overall team to ensure all players in a squad have experienced a similar training load. Therefore, we would recommend that both relative and absolute measures are reported in future research.

### 4.2. Forward and back comparison in different levels of competition

The trend of the lower levels of competition covering more total distance generally persisted when data was separated into forward and backs, especially for semi-professional (Div 2) players who had significantly higher relative total distance measures compared to all other levels of competition. In the running data, there was a tendency for amateur level backs to record significantly less overall running and high intensity running distances than the higher competition levels. Amateur level forwards also recorded significantly less high intensity running distance than higher competition levels. Once again, the highest running distances were recorded in semi-professional (Div 2), with backs and forwards running more than all other levels of competition. However, there was minimal difference in high intensity running measurements for back and forwards between semi-professional (Div 2) and the higher levels of competition data.

The relative total distance covered by amateur forwards and backs (65 m.min^−1^ and 75 m.min^−1^) in this study were similar to data on university forwards and backs in research by Read et al. (23) (67 m.min^−1^ and 71 m.min^−1^ respectively). The outcome that amateur competition typically had lower running demands compared to higher levels of competition was also similar to the findings by Read et al. (18, 23), who generally found increased running loads as players progressed through age groups to university level competition. Overall, the range of relative total distance measures in all competitions (62 m.min^−1^ and 83 m.min^−1^, forwards and backs) were similar to the range of values reported in a systematic review of GPS rugby research by Bridgeman and Gill (2), although the highest values from the semi-professional (Div 2) data (71 m.min^−1^ and 83 m.min^−1^, forwards and backs) were in the upper range of the measurements presented in the review.

### 4.3. Forward and back positions comparison in different levels of competition

Amateur level front and second row players covered significantly more distance at lower intensities but typically ran less at high intensity than semi-professional or professional players. Club level back rowers also covered similar or less total distance compared to other levels and did significantly less high intensity running compared to higher competition levels. Consequently, amateur forwards may struggle with the added pace of the game when transitioning to higher level competitions. However, it should be noted that the magnitude of the differences between the amateur and semi-professional and professional players ranged from 0 to 3 m.min^−1^ for high intensity running measures. While the majority of these differences were statistically significant, it is not known whether differences of this size would impact on the performance of players during 60 or more minutes of rugby. In contrast, based on our data, semi-professional (Div 2) forwards would presumably not struggle with the running loads of the higher semi-professional (Div 1) or professional competitions, as they typically recorded similar or significantly greater running values during games. Whereas semi-professional (Div 1) second rowers may find professional rugby significantly faster with relatively more running and high intensity running occurring at this level of competition.

Amateur outside backs covered significantly less running and high intensity running compared to higher competition levels. Therefore, these players may struggle when transitioning to higher level competitions as the magnitude of the differences for these measures ranged from 5 to 9 m.min^−1^ and 3–5 m.min^−1^ respectively. Whereas the differences between amateur inside backs and semi-professional and professional players for running measures were typically minimal and non-significant. The differences between amateur half backs and other levels were less clear cut, where the amateur half backs typically ran significantly less than semi-professional (Div 2) and professional players but not semi-professional (Div 1) half backs.

Based on our findings for back and forward positions in the different levels of competition, running demands cannot be assumed to increase with higher levels of competition due to the variability in running measures in playing positions within a team and the levels of competitions. For example, front row forwards and inside backs have similar running loads at all levels of competition vs. amateur back row forwards and outside backs who typically recorded significantly lower running loads compared to higher competition players. Therefore, when transitioning players, the specificity of the running demands of each position needs to be considered when preparing a player for another level of competition as suggested in a review by Bridgeman and Gill (2). As in our study, Tierney et al. (21) found that lower-level competition players recorded greater relative total distances per game in different European professional competitions (21). However, Tierney et al. (21) also found that relative running and high-speed running were higher in the lower levels of professional rugby competitions. In contrast, as discussed earlier, our study found considerable variation in running distances within teams and between competitions. The difference between our research and Tierney et al. (21) could be due to a wide range of factors such as coaching strategies, training methods and weather conditions (19). Irrespective of the differences, our findings align with Tierney and colleagues' assertion that a qualitative perception or assumption that lower levels of competition are less demanding than higher levels of competition are not supported.

### 4.4. Loading and rehabilitation

The position-and-competition specific findings in this research could be useful when designing rehabilitation programs for an injured rugby player. In return to sport, training load is generally progressed from rehabilitation for an injury, to non-contact running and then on to specific rugby training programmes based on positional demands (24–28). Our findings provide data about the positional demands for the different levels of competition, which could be useful for a practitioner, as there is no general consensus on return to play in rugby in musculoskeletal injuries, with return to sport decision-making being under reported, having a lack of standardization and clear criteria (24–28). Therefore, objective data about running load could provide useful information in the design of return to play programmes for players. In terms of practical application, practitioners should be cautious in assuming that lower-level competitions have lower running demands. As previously stated, we did not find a clear-cut continuum where increased level of competition increased running demands, for example, semi-professional (Div 2) players typically ran more distance than all other levels. Therefore, it cannot be assumed that running load is less in lower levels of competition, as we found loading is position specific and there is considerable variation within a competition level. For example, amateur level half-or-outside backs covered significantly less distance in all measures compared to a professional player, however, there was minimal difference between the running demands of amateur inside backs and the other levels of competition. Therefore, the running load of each specific position in a competition needs to be considered individually as part of a return to play programme for a player. Subsequently, the practice of professional players sometimes returning to play after injury *via* amateur club level competition needs to be undertaken with caution and preferably with live or real time GPS to monitor loading. Additionally, practitioners that do decide to bring injured players back to play through lower rugby levels, considering the demands for some positions may be similar, the practitioner may need to consider decreasing total match/game time in these lower-level games.

### 4.5. Limitations

A limitation of this study is that acceleration, deceleration, and impact data was not recorded. It is likely that there could be considerable variation in these measures in the amateur, semi-professional and professional competitions based on previous research findings on rugby for these variables. For example, Tierney et al. found collision frequency and intensity increased when progressing to higher levels of competition. Subsequently, we cannot discount that while the running demands of some positions in our study such as inside backs may be similar across competitions, it is possible there may be considerable differences in the loads experienced by players across competitions from impacts and collisions in a game. Additionally, our study did not quantify the types of movements occurring during games such as tackle and scrummage, coaching strategies, weather and fitness levels of players, all of which could influence players and a team's performance. Due to the applied nature of the research and use of convenience samples, different manufacturers’ GPS units were used and the number of competition games per team varied (range: 9–17), we cannot exclude the possibility that differences between teams are due to the use of different GPS units or that outcomes may lack external validity due to the small sample of teams used in the research (i.e., two teams in the 2017 rugby season and two teams in the 2019 season). A potential confounding variable is also the amount of time players were on the field. Attempts were made to control for this variability by only including players who played 60 min or more and using the relative m.min^−1^ measure. However, it is possible there may have been variation in playing time between positions and competitions, as the average time for teams ranged between 79 min-83 min. Future research should account for the specific time played in each position. Finally, because the data was not collected in the same season (there was a 2-year gap between amateur and semi-professional (Div 2) data collection and semi-professional (Div 1) and professional), we suggest caution should be taken when interpreting the data as changes in variables between seasons such as pitch and environmental conditions may introduce confounding issues in the data.

## 5. Conclusion

There were typically minimal differences in the running distance covered in a game between amateur level front and second row forwards, and inside backs compared to players in these positions at higher levels of competition. Therefore, club players in these positions would probably cope well when transitioning to higher level rugby competitions from a running perspective. In contrast, amateur outside backs and back row forwards may find the increased pace of higher levels of competition difficult due to typically covering significantly less running and high intensity running distances in amateur club games. Differences for the half back were variable, where amateur level players had similar measures as semi-professional (Div 1) players but typically covered significantly less running distances than semi-professional (Div 2) and professional half backs. Based on the contrasting and variable findings for running demands in the different playing positions, it cannot be assumed that amateur or similarly semi-professional (Div 2) (second lowest team) positions have lower running demands than higher competitions [semi-professional (Div 1) and professional]. Therefore, the specificity of running demands in a position and competition need to be considered for each player when transitioning players between competitions. Similarly, when a player is returning to rugby after an injury, the practice and perception of returning a professional player to amateur club rugby due to running loads being lower may be flawed, as we found considerable position variation in running demands within-and-between competition levels.

## Data Availability

The raw data supporting the conclusions of this article will be made available by the authors, without undue reservation.

## References

[1] Coughlan G Green B Pook P Toolan E O'Connor S. Physical game demands in elite rugby union: a global positioning system analysis and possible implications for rehabilitation. J Orthop Sports Phys Ther. (2011) 41(8):600–5. 10.2519/jospt.2011.3508 21654094

[2] Bridgeman LA Gill ND. The use of global positioning and accelerometer systems in age-grade and senior rugby union: a systematic review. Sports Med Open. (2021) 7(1):15. 10.1186/s40798-021-00305-x 33616786 PMC7900280

[3] Coutts A Duffield R. Validity and reliability of GPS devices for measuring movement demands of team sports. J Sci Med Sport. (2010) 13(1):133–5. 10.1016/j.jsams.2008.09.015 19054711

[4] Cummins C Orr R O'Connor H West C. Global positioning systems (GPS) and microtechnology sensors in team sports: a systematic review. Sports Med. (2013) 43(10):1025–42. 10.1007/s40279-013-0069-2 23812857

[5] Jones M West D Crewther B Cook C Kilduff L. Quantifying positional and temporal movement patterns in professional rugby union using global positioning system. Eur J Sports Sci. (2015) 15:488–96. 10.1080/17461391.2015.1010106 25675258

[6] Austin D Gabbett T Jenkins D. The physical demands of super 14 rugby union. J Sci Med Sport. (2011) 14(3):259–63. 10.1016/j.jsams.2011.01.003 21324741

[7] Cahill N Lamb K Worsfold P Heady R Murray S. The movement characteristics of English premiership rugby union. J Sports Sci. (2013) 31:229–37. 10.1080/02640414.2012.727456 23009129

[8] Cunniffe B Proctor W Baker J Davies B. An evaluation of the physiological demands of elite rugby union using global positioning system tracking software. J Strength Cond Res. (2009) 23:1195–203. 10.1519/JSC.0b013e3181a3928b 19528840

[9] Deutsch M Kearney G Rehrer N. Time-motion analysis of professional rugby union players during match-play. J Sports Sci. (2007) 25:461–72. 10.1080/02640410600631298 17365533

[10] Duthie G Pyne D Hooper S. Applied physiology and game analysis of rugby union. Sports Med. (2003) 33:973–91. 10.2165/00007256-200333130-00003 14606925

[11] Lacome M Piscione J Hager JP Bourdin M. A new approach to quantifying physical demand in rugby union. J Sports Sci. (2014) 32:290–300. 10.1080/02640414.2013.823225 24016296

[12] Lindsay A Draper N Lewis J Gieseg S Gill N. Positional demands of professional rugby. Eur J Sports Sci. (2015) 16:480–7. 10.1080/17461391.2015.1025858 25830235

[13] Quarrie K Hopkins W Anthony M Gill N. Positional demands of international rugby union: evaluation of player actions and movements. J Sci Med Sport. (2013) 16:353–9. 10.1016/j.jsams.2012.08.005 22975233

[14] Roberts S Trewartha G Higgitt R El-Abd J Stokes K. The physical demands of elite English rugby union. J Sports Sci. (2008) 26:825–33. 10.1080/02640410801942122 18569548

[15] Dubois R Paillard T Lyons M McGrath D Maurelli O Prioux J. Running and metabolic demands of elite rugby union assessed using traditional. Metabolic power, and heart rate monitoring methods. J Sports Sci Med. (2017) 16:84–92. 28344455 PMC5358036

[16] King D Cummins C Hume P Clark T Pearce A. Physical demands of amateur senior domestic rugby union players over one round of competition matches in New Zealand assessed using heart rate and movement analysis. Inter J Sports Sci Med. (2018) 2:066–71.

[17] Read D Jones B Phibbs P Roe G Darrall-Jones J Weakley J The physical characteristics of match-play in English schoolboy and academy rugby union. J Sports Sci. (2017) 36(6):645–50. 10.1080/02640414.2017.1329546 28514202

[18] Read D Jones B Phibbs P Roe G Darrall-Jones J Weakley J Physical demands of representative match-play in adolescent rugby union. J Strength Cond Res. (2017) 31:1290–6. 10.1519/JSC.0000000000001600 27548792

[19] Takamori S Hamlin MJ Kieser DC King D Hume P Yamazaki T Senior club-level rugby union Player's Positional movement performance using individualized velocity thresholds and accelerometer-derived impacts in matches. J Strength Cond Res. (2022) 36:710–6. 10.1519/JSC.0000000000003523 32168074

[20] New Zealand Rugby Union. New Zealand Rugby competitions. New Zealand Rugby (2019). [Online]. Available from: https://www.nzrugby.co.nz/teams-and-competitions/.

[21] Tierney P Blake C Delahunt E. Physical characteristics of different professional rugby union competition levels. J Sci Med Sport. (2021) 24:1267–71. 10.1016/j.jsams.2021.05.009 34144858

[22] Reardon C Tobin D Delahunt E. Application of individualized speed thresholds to interpret position specific running demands in elite professional rugby union: a GPS study. PLoS One. (2015) 10:e0133410. 10.1371/journal.pone.0133410 26208315 PMC4514747

[23] Read D Weaving D Phibbs P Darrall-Jones J Roe G Weakley J Movement and physical demands of school and university rugby union match-play in England. BMJ open Sport Exerc Med. (2017) 2:e000147. 10.1136/bmjsem-2016-000147 28879027 PMC5569272

[24] Beardmore A Handcock P Rehrer N. Return-to-play after injury: practices in New Zealand rugby union. Phys Ther Sport. (2005) 6:24–30. 10.1016/j.ptsp.2004.04.002

[25] Erickson L Sherry M. Rehabilitation and return to sport after hamstring strain injury. J Sport Health Sci. (2017) 6:262–70. 10.1016/j.jshs.2017.04.001 30356646 PMC6189266

[26] Hurley E Withers D King E Franklyn-Miller A Jackson M Moran R. Return to play after patellar tendon autograft for primary anterior cruciate ligament reconstruction in rugby players. Orthop J Sports Med. (2021) 9(5):23259671211000460. 10.1177/2325 PMC8114267 34017876

[27] Sclafani M Davis C. Return to play progression for rugby following injury to the lower extremity: a clinical commentary and review of the literature. Int J Sports Phys Ther. (2016) 11:302–20. 27104062 PMC4827372

[28] Reid C Cowman J Green B Coughlan G. Return to play in elite rugby union: application of global positioning system technology in return-to-running programs. J Sports Rehabil. (2013) 22(2):122–9. 10.1123/jsr.22.2.122 23238265

